# The Unaddressed Behavioral Health Aspect During the Coronavirus Pandemic

**DOI:** 10.7759/cureus.7351

**Published:** 2020-03-21

**Authors:** Henry K Onyeaka, Shaheer Zahid, Rikinkumar S Patel

**Affiliations:** 1 Epidemiology, Harvard School of Public Health, Boston, USA; 2 Psychiatry, Saint James School of Medicine, Park Ridge, USA; 3 Psychiatry, Griffin Memorial Hospital, Norman, USA

**Keywords:** corona virus, pandemic, behavioral disorder, psychiatric co-morbidity, social and behavioral epidemiology, covid-2019, covid-19

## Abstract

The 2019 novel coronavirus (2019-nCoV) pneumonia has been declared a pandemic, citing more than 118,000 cases of the coronavirus illness in more than 110 countries and territories around the world. Public health emergencies have been demonstrated to have an impact on the behavioral health of the affected population as they may experience fear, anxiety, anger and post-traumatic stress disorder as consequences of their experiences. These effects may persist among affected individuals long after the outbreak has been controlled. To date, data on the behavioral distress and psychiatric morbidity of those suspected or diagnosed with the 2019-nCoV and their treating health professionals are lacking. Although the Centers for Disease Control and Prevention (CDC) has outlined some behavioral health guide for affected individuals, how best to respond to psychological challenges during the crisis is not known. There is an urgent need to provide robust and timely psychosocial support in the face of such an outbreak.

## Editorial

The 2019 novel coronavirus (2019-nCoV) pneumonia, which originated in the Hubei province in China at the end of 2019, has gained intense attention nationwide and globally. In the United States, since the first case was detected in Washington, DC, in mid-January 2020, the virus has continued to spread. As of March 19, 2020, a total of 10,442 confirmed cases have been reported in 54 jurisdictions (50 states, District of Columbia, Puerto Rico, Guam and US Virgin Islands) and has resulted in 150 deaths [1]. On March 11, the World Health Organization (WHO) declared 2019-nCoV a pandemic, citing more than 118,000 cases of the coronavirus illness in more than 110 countries and territories around the world and the sustained risk of further global spread.

While the exact origin remains largely unknown, the virus has been shown to cause respiratory illness ranging from mild to severe and is spread via human to human transmission. At the moment, the therapeutic strategies to deal with the infection are only supportive and preventive. Based on the available information regarding the virus, upgraded quarantine and isolation measures have been suggested to resist the spread of the virus. The Centers for Disease Control and Prevention (CDC) has issued detailed guidelines and recommendations to stem community spread of the virus.

Public health emergencies have been demonstrated to have an impact on the behavioral health of the affected population [2]. Individuals and their families with confirmed or suspected 2019-nCoV may experience fear, anxiety, anger and post-traumatic stress disorder as consequences of their experiences. These effects may persist among affected individuals long after the outbreak has been controlled. Furthermore, contact tracing and the mandatory quarantine isolation for two weeks, which is a crucial part of the public health responses to the 2019-nCoV pneumonia outbreak, could be a precursor for increased psychological distress such as post-traumatic stress disorder, anxiety and anger among suspected or confirmed cases [3,4]. Also, the estimated global economic shutdown and recession is expected to heighten fears and anxiety.

Health professionals, especially those directly caring for people with confirmed or suspected 2019-nCoV pneumonia, are susceptible to both high risk of infection and behavioral health distress. The literature has documented high levels of psychological distress among healthcare workers in previous outbreaks. They may experience fear of contracting and spreading the virus to their families, friends or colleagues. In a cross-sectional study by Wu et al., the healthcare workers who had been quarantined, or worked in the high-risk location such as severe acute respiratory syndrome (SARS) wards, or had friends or close relatives who contracted SARS, were significantly more likely to have high post-traumatic stress symptom levels than those without these exposures [5].

To date, data on the behavioral distress and psychiatric morbidity of those suspected or diagnosed with the 2019-nCoV and their treating health professionals are lacking. Although the CDC has outlined some behavioral health guide for affected individuals, how best to respond to psychological challenges during the crisis is not known. There is an urgent need to provide robust and timely psychosocial support in the face of such an outbreak.

Therefore, using lessons learned from prior epidemics like SARS, Middle East respiratory syndrome and the ongoing 2019-nCoV in China, some potential methods to mitigate the psychosocial impact of the pandemic should be emphasized. First, it is the provision of clear, accurate and updated information about the virus to both health workers and patients in order to allay their anxiety and fear. Second, robust behavioral health services should be deployed to deliver behavioral health support to patients and health workers with additional training of more healthcare professionals in psychological first aid delivery to the high-risk population. Lastly, given the present challenge of cross-infection from the onsite and face-to-face delivery of healthcare, telehealth and other remote forms of behavioral health delivery should be encouraged.

The current 2019-nCoV outbreak may stretch the already limited behavioral health services, and prompt measures must be instituted to avert the potential acute and long-term psychological sequela that may ensue.
